# Predicting resectability after neoadjuvant chemotherapy for patients with borderline resectable pancreatic cancer: a single center, retrospective trial

**DOI:** 10.3389/fonc.2025.1602933

**Published:** 2025-09-19

**Authors:** Florian Schepp, Sebastian Hempel, Felix von Bechtolsheim, Felix Merboth, Olga Radulova-Mauersberger, Christian Teske, Nicolas Mibelli, Lena Seifert, Jürgen Weitz, Marius Distler, Florian Oehme

**Affiliations:** ^1^ Department of Visceral, Thoracic and Vascular Surgery, University Hospital and Faculty of Medicine Carl Gustav Carus, Technische Universität Dresden, Dresden, Germany; ^2^ National Center for Tumor Diseases (NCT/UCC), Dresden, Germany; ^3^ German Cancer Research Center (DKFZ), Heidelberg, Germany; ^4^ Faculty of Medicine and University Hospital Carl Gustav Carus, Technische Universität Dresden, Dresden, Germany; ^5^ Helmholtz-Zentrum Dresden - Rossendorf (HZDR), Dresden, Germany

**Keywords:** prediction of resectability, carbohydrate antigen 19-9, CA19-9, pancreatic cancer, resectability, neoadjuvant chemotherapy

## Abstract

**Background/Objectives:**

Pancreatic ductal adeno carcinoma (PDAC) in its borderline resectable (BR) stage often limits the possibility of complete resection, currently the only potential path to a cure. Neoadjuvant chemotherapy seeks to downsize tumors, thereby increasing the chances of achieving an R0 resection. However, accurately predicting resectability following such treatment remains challenging. This study aims to identify and evaluate potential biomarkers that may improve preoperative assessment of tumor resectability during exploratory laparotomy, thereby minimizing the incidence of futile surgical interventions and their associated morbidity in patients with non-resectable malignancies.

**Methods:**

We conducted a retrospective analysis of all patients who underwent exploratory laparotomy following neoadjuvant chemotherapy at the Department of Visceral, Thoracic, and Vascular Surgery, University Hospital Dresden, between 2011 and 2022. Employing a propensity score matching, we compared patients with resectable and unresectable pancreatic cancer. The primary endpoint was to evaluate preoperative parameters for predicting resectability status.

**Results:**

This study included initially 134 patients with neoadjuvant treated BR PDAC who underwent exploratory laparotomy. Among them, 100 (74.6%) underwent curative intended resection, and 34 (25.4%) had an explorative laparotomy only. After Propensity Score Matching we found that a pre-chemotherapy CA 19–9 Value < 450 U/ml (OR 2.9; 95% CI: 1.04 – 8.53, p = 0.04), a pre-operative CA19–9 value of < 105 U/ml (OR 13.9; 95% CI: 3.88 – 49.7, p = 0.001), and a pre - to post-chemotherapy CA19–9 ratio of ≤ 15% (OR 9; 95% CI: 2.48 – 32.7, p = 0.001) raised the odds for resectability. Further, if the combination of a preoperative CA 19-9 < 105 U/ml and a pre - to post-chemotherapy CA19–9 ratio of < 15% was present, the resectability rate increased up to 93%, compared to 30% when both parameters were above the threshold. This specific constellation was a significant predictor (OR 63; 95% CI: 7.84 – 506, p = 0.001) for curative resectability.

**Conclusions:**

Our data highlight not only the significant role of the preoperative CA19–9 value and the pre- to post-chemotherapy CA19–9 ratio but, even more importantly, emphasize the critical impact of the combination of these two parameters on the resectability of BR-PDAC.

## Introduction

The prevalence of PDAC is expected to rise significantly in the coming decades, with projections indicating a significant rise in the number of patients diagnosed with the disease (1, 2). The current screening methods for PDAC are limited, often leading to diagnoses at advanced stages, such as BR PDAC (3, 4). Consequently, the mortality rate for pancreatic cancer is expected to nearly double by 2040 compared to current levels (4). Historically, the only potential curative treatment for pancreatic cancer has been radical surgery with the goal of achieving an R0 resection (5, 6). However, pancreatic surgery is associated with high morbidity and mortality rates and can be exceedingly taxing for patients (7). Adjuvant chemotherapy has long been a staple in the treatment of pancreatic cancer, demonstrating a substantial improvement in survival rates (8). Despite this, the aggressive nature of the disease and its treatment results in only about 50% of patients receiving adjuvant chemotherapy after surgery, and a mere 7% completing the full course (9). This low completion rate is attributed to postoperative deterioration, disease progression, and the high complication rates linked with radical surgery (7). To address these challenges, neoadjuvant chemotherapy (naCTx) has gained increasing importance especially for BR PDAC (10–12). Neoadjuvant therapy aims to downsize tumors before surgery, potentially reducing the necessity for vascular resection (9, 13). A notable study in this field is the Dutch multicenter phase III PREOPANC trial, which randomized 246 patients with resectable, borderline resectable and locally advanced tumors to receive either neoadjuvant chemoradiotherapy or immediate surgery (14). With a median follow-up of 59 months, the study demonstrated that neoadjuvant therapy led to a significantly better overall survival in both borderline and locally advanced resectable tumors (14). Additionally, the multicenter ESPAC-5 trial demonstrated a significant improvement in one-year survival rates following naCTx with either FOLFIRINOX or gemcitabine-based regimens (15). These findings endorse the use of neoadjuvant therapy to reduce tumor stage, lessen the extent of vascular resection, and enhance R0 resection rates (16). However, the exact benefits of naCTx across all stages of pancreatic cancer remain incompletely understood. Imaging techniques, such as CT or MRI, can predict tumor resectability with an accuracy of approximately 80% (3, 17). Nonetheless, 10 – 25% of patients initially deemed resectable based on preoperative clinical assessment and imaging ultimately require intraoperative abandonment of surgery due to unforeseen distant metastases or disseminated peritoneal carcinomatosis (18, 19). This underscores the necessity for additional evidence to better select patients and avoid unnecessary exploration which is associated with substantial increase in complications and increase healthcare costs (20). Consequently, the assessment of surgical resectability is expected to become increasingly critical in the future. Our retrospective propensity score-matched analysis of patients with BR PDAC treated with naCTx aims to identify those most likely to benefit from surgical exploration following chemotherapy. This study highlights the significance of carbohydrate antigen 19-9 (CA19-9), a key biochemical marker in PDAC, as a predictive tool for selecting candidates for curative R0 resection.

## Materials and methods

This article was written following the strengthening for the reporting of observational studies in epidemiology Statement (STROBE) (21). The experimental protocol of the study was approved by the local ethics committee of the TU Dresden (decision number EK 576122019).

### Cohort definition and inclusion criteria

All patients with BR PDAC who underwent naCTx from 01/2011 to 12/2022 were included in this study. In deviation from the German S3 guidelines for pancreatic cancer, borderline resectability was defined at our institution as any radiologically detected vascular involvement of either the celiac trunk, superior mesenteric artery (SMA) or hepatic artery (22). This definition was applied because current literature supports the fact that arterial resection can be performed safely in high volume centers and improve patient survival in selected cases (23, 24). At our center, arterial resections were routinely performed using venous interposition grafts or end-to-end anastomoses, with the primary aim of achieving R0 resection. Staging was performed through contrast-enhanced computed tomography (CT) and confirmed histologically via CT-guided transabdominal or transgastric/transduodenal biopsy. Patient cases were discussed at an interdisciplinary tumor board, which recommended a neoadjuvant chemotherapy regimen typically comprising six to eight preoperative CTx-cycles. Chemotherapy regimen (FOLFOXIRI/Gemcitabine (+)) was decision of the interdisciplinary tumor board.

Following four cycles of chemotherapy, an interim contrast enhanced CT assessment was performed. In cases of stable imaging findings, chemotherapy was continued. In the event of local tumor progression in contrast enhanced CT, the neoadjuvant treatment regimen was adjusted. Newly identified metastases were histologically confirmed, and the patient was excluded from this analysis with progressive disease. Patients with stable tumor findings or evident therapy response continued their treatment. After completion of the full chemotherapy protocol, a comprehensive evaluation was conducted, including another contrast enhanced CT and a full laboratory workup with tumor marker analysis. Based on CT findings, patients with stable disease or tumor regression were scheduled for exploratory laparotomy approximately 6–8 weeks after completion of neoadjuvant chemotherapy, regardless of the CA19–9 dynamic.

### Surgical approach and cohort definition

Following abdominal exploration and confirmation of the absence of metastases, technical tumor resectability was assessed. Tumors were classified as resectable (cohort 1: nCTxR) if infiltrated vascular structures, both central and peripheral, could be reconstructed. Tumors were deemed unresectable (cohort 2: nCTxU) if distant metastases (e.g., peritoneal carcinomatosis, liver metastases) were present or if arterial reconstruction was deemed impossible.

### Patient data

Patient data included standard basic patient data (e.g. age, sex, BMI) as well as laboratory values prior to chemotherapy and surgery. We further analyzed extent of surgery, postoperative complications, and length of hospital stay. These details were extracted from the clinical information system. Additional parameters, such as tumor location, type and duration of neoadjuvant chemotherapy, tumor board recommendations, and histological findings, were sourced from the tumor documentation system. CA19–9 levels were measured prior to the initiation of chemotherapy and again before surgery (on the day of hospital admission).

### Matching

**Figure 1:**
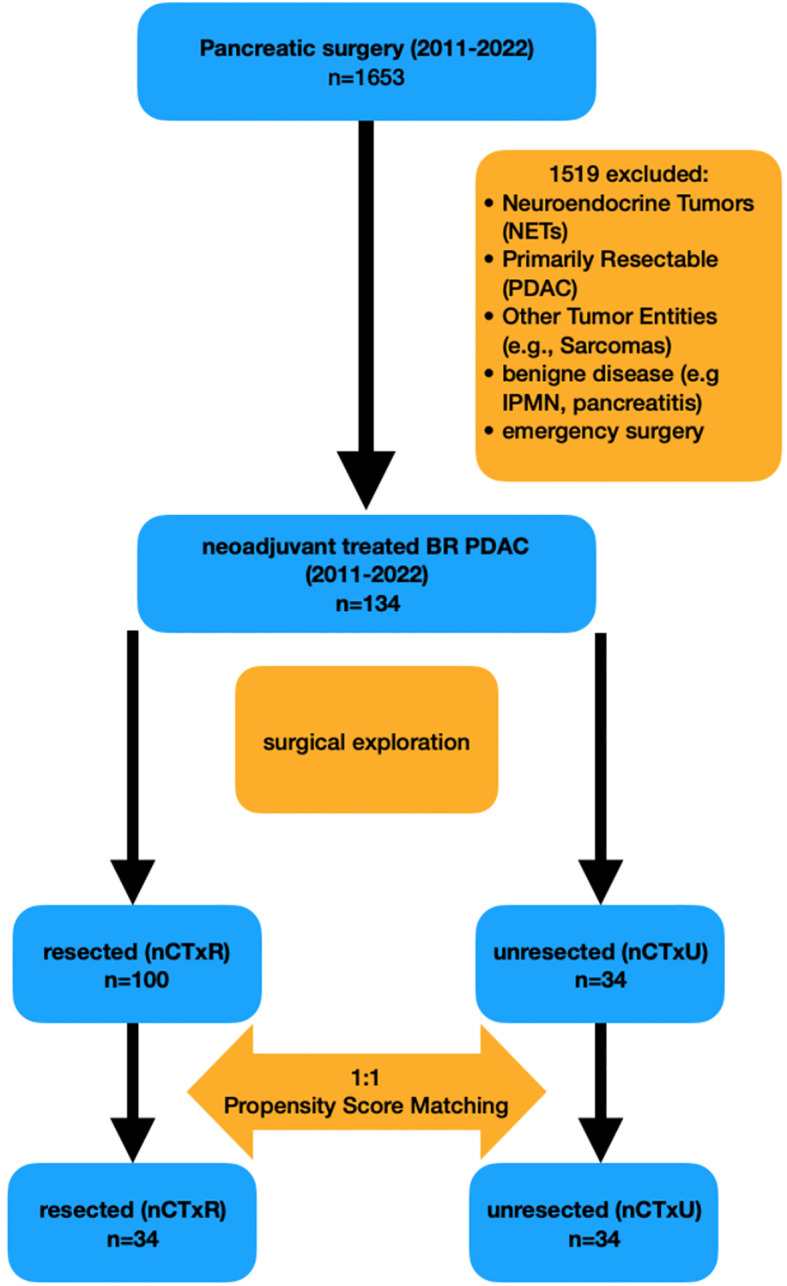
Cases included in and excluded from the study. BR borderline resectable.

The initial database included 1653 patients who had undergone explorative laparotomy with the aim to perform a pancreatic surgery. Patients with neuroendocrine Tumor, primary resectable PDAC, other tumor entities or benign disease were excluded. In total, 134 Patients with BR PDAC and explorative laparotomy after naCTx were included. Among these, 100 patients were classified as resectable with curative intent (nCTxR), while 34 underwent exploratory laparotomy only (nCTxU). Subsequently, nCTxR and nCTxU patients were paired employing a propensity score matching (nearest neighbor method). After matching, we checked for any remaining imbalances using a standardized mean deviation analysis. Finally, 34 patients remained in the analysis in both groups. **Figure 1** illustrates the distribution of patients across both groups.

### Endpoint

The primary endpoint of the study was to define a cut-off value for CA 19–9 at which the likelihood for a curative intended resection in BR PDAC patients significantly increases. The pre-to-post-chemotherapy ratio was calculated by dividing the CA19–9 value after naCTx by the CA19–9 value measured prior to the initiation of naCTx.

Secondary endpoints were defined overall survival, complication rate and length of hospital stay.

### Statistical methods

The statistical analysis was conducted using SPSS software, version 27 (IBM Corp., Armonk, NY, USA). Firstly, normality distribution of the continuous data was tested employing the Kolmogorov-Smirnov test and by reviewing frequency distributions. To assess the equality of variance among groups, Levene’s test was utilized. To compare baseline characteristics, we conducted analyses for categorical variables using either the chi-square test or Fisher’s exact test, and for continuous variables, we applied the Student’s t-test or the Mann-Whitney U test as suited. A p-value below 0.05 was set as the significance cutoff for the study. Missing data were assumed to be missing completely at random.

We performed a propensity score-matched analysis between resected (nCTxR) and unresected (nCTxU) patients using the nearest neighbor method at a 1:1 ratio. The propensity score deviation width was set to a threshold of < 0.2. Variables used for matching included age, neoadjuvant chemotherapy protocol, BMI, ASA score, preoperative biliary drainage, and insulin-dependent diabetes mellitus (IDDM). To detect residual imbalances after matching, we employed a standardized mean deviation analysis.

Finally, we defined cutoff values for each laboratory parameter performing a receiver operating characteristics (ROC) curve and assessing the area under the curve (AUC). Subsequently, we created binary variables from the laboratory parameters to assess the impact of these cutoffs on resectability (see **Supplementary Table 1**). Univariate and multivariate analyses were performed on the significant and/or clinically relevant parameters.

## Results

### Pre-matching basic patient characteristics

Of 134 patients, tumor resection was performed in 100 patients (74.6%), while 34 patients (25.4%) deemed unresectable. Overall, 78 patients (58.3%) in the study cohort were women, and 40 patients (40%) in the nCTxR group were men (p = 0.47). The median age was 66 years (IQR 61 - 69.7 years) in the nCTxR and 65.5 years (IQR 59.5 – 72.2 years) in the nCTxU (p = 0.69). A total of 46 (34.3%) patients received a preoperative biliary drainage with 38 (38%) patients in the nCTxR and 8 (23.5%) in the nCTxU cohort (p = 0.42), respectively. (**Table 1**)

**Table 1:** Basic patient characteristics pre- and after matching cohort.

	Pre-matching			Post-matching		
	nCTxR	nCTxU	P	nCTxR	nCTxU	P
patients [n (%)]	100 (74.6)	34 (25.4)		34 (50)	34 (50)	
Sex [n (%)]						
m	40 (40)	16 (47)	0.47	14 (41.2)	16 (47.1)	0.63
w	60 (60)	18 (52.9)		20 (58.8)	18 (52.9)	
age (years; (IQR))	66 (61-69.7)	65.5 (59.5 – 72.2)	0.69	66 (59 – 68.5)	64.5 (56.5 – 68.25)	0.82
BMI (kg/m²; (IQR))	23.5 (21.5 – 26)	23.4 (21.7 – 25.1)	0.55	25.3 (23.4 – 26.4)	23.4 (21.7 – 25.2)	0.79
smoking [n (%)]	19 (19)	2 (5.9)	0.17	9 (26.5)	2 (5.9)	0.06
alcohol [n (%)]	8 (8)	0 (0)	0.13	3 (8.8)	0 (0)	0.13
ASA score [n (%)]						
1	3 (3)	3 (8.8)	0.22	1 (2.9)	3 (8.8)	0.53
2	42 (42)	10 (29.4)		17 (50)	10 (29.4)	
3	52 (52)	12 (35.3)		14 (41.2)	12 (35.3)	
4	3 (3)	2 (5.9)		2 (5.9)	2 (5.9)	
diabetes [n (%)]	41 (41)	14 (41.2)	0.31	15 (44.1)	14 (41.2)	0.55
insulin-dependent diabetes (IDDM) [n (%)]	25 (23)	9 (26.5)	0.34	10 (29.4)	9 (26.5)	0.73
preoperative biliary drainage [n (%)]	38 (38)	8 (23.5)	0.42	10 (29.4)	8 (23.5)	0.99

### Post-matching basic patient characteristics

After matching, each group consisted of 34 patients. The median age in the nCTxR group was 66 years (IQR 59 – 68.5 years), and nCTxU group 64.5 years (IQR 56.5 – 68.25 years) (p = 0.82). Through matching, we improved the comparability of patients with preoperative biliary drainage, which was performed in 10 patients (29.4%) in the nCTxR group and 8 patients (23.5%) in the nCTxU group (p = 0.99). Additionally, matching reduced the proportion of patients with an ASA score of 3 to 14 (41.2%) in the nCTxR group, compared to 52 patients (52%) prior to matching. Further, the comparability between the groups was improved. After matching, the BMI was 23.4 kg/m² (IQR 21.7 – 25.2 kg/m²) in the nCTxU and 25.3 kg/m² (IQR 23.4 – 26.4 kg/m²) in the nCTxR (p = 0.79). (**Table 1**)

### Tumor location and basic oncological parameters

The most common tumor location was in the head of the pancreas, found in 49 patients (72.1%). In the nCTxU group 28 patients (82.4%) had a tumor located in the pancreatic head, while 21 patients (61.7%) in the nCTxR group had a tumor in this region. Five (14.7%) patients in the nCTxR group had a tumor in the pancreatic tail. The most frequent infiltrated vessels, as demonstrated by computed tomography, were the superior mesenteric artery (SMA) (n=25, 36.7%), the superior mesenteric vein (SMV) (n=26, 38.2%), and the portal vein (n=22, 32.3%). The most administered chemotherapy regimen was FOLFOX/IRI, used in a total of 39 patients (57.4%), followed by Gemcitabine ± Nab-Paclitaxel/Cisplatin in 29 patients (42.6%). Results shown in **Table 2**.

**Table 2:** Basic oncological and therapeutic parameters.

	Overall	nCTxR	nCTxU	P
Tumor localization [n (%)]				
Head	49 (72.1)	21 (61.8)	28 (82.4)	0.14
Corpus	14 (20.6)	8 (23.5)	6 (17.6)	
Tail	5 (7.3)	5 (14.7)	0	
Radiological BR PDAC [n (%)]				
Truncus coeliacus	16 (23.6)	8 (23.5)	8 (23.5)	0.4
SMA	25 (36.7)	9 (26.5)	16 (47.1)	
SMV	26 (38.2)	15 (44.1)	11 (32.4)	
Portal vein	22 (32.3)	15 (44.1)	7 (20.5)	
Hepatic artery	19 (27.9)	11 (32.4)	8 (23.5)	
Metastases [n (%)]				
Liver	14 (20.6)	1 (2.9)	13 (55.9)	**n.a**
Nodal	2 (2.9)	1 (2.9)	1 (2.9)	
Pulmonal	1 (1.5)	0 (0)	1 (2.9)	
Peritoneal	3 (4.4)	0 (0)	3 (8.8)	
Multiple	3 (4.4)	0 (0)	3 (8.8)	
Chemotherapy protocol [n (%)]				
Gemcitabine (+)	29 (42.6)	14 (41.2)	15 (44.2)	0.89
FOLFOX/IRI	39 (57.4)	20 (58.8)	19 (55.8)	
PreCTx tumor board assessment [n (%)]				
curative	51 (75)	32 (94.1)	19 (55.8)	**0.001**
palliative	15 (22.1)	2 (5.6)	13 (38.2)	
Time from diagnosis to CTx ([days] (IQR))	30 (21.25 – 48)	33.5 (21.5 – 46.75)	30 (20.75 – 49.25)	0.61
Operation [n (%)]				
PPPD/Whipple		15 (44.1)		**n.a**
Distal Pancreatectomy		5 (14.7)		
Pancreatectomy		14 (41.2)		
Only exploration			18 (52.9)	
Gastroenterostomy (GE)			6 (17.7)	
Biliodigestive anastomosis (BDA)			2 (5.9)	
GE + BDA			8 (23.5)	
Resected vessels/Reconstructed vessels [n (%)]				
Coeliac trunc		10 (29.4)		**n.a**
Hepatic artery		5 (14.7)		
SMA		4 (11.8)		
SMV		4 (11.8)		
Portal veine		18 (52.9)		
R0 resection [n (%)]		29 (85.3)		

For a clearer overview, we combined all patients treated with gemcitabine-based regimens. Many of these patients received gemcitabine in combination with additional agents (e.g nab-paclitaxel, capecitabine, Cisplatin). Bold values indicate statistical significance (p < 0.05).

### Type of resection in the nCTxR cohort

In the nCTxR cohort, pancreatic head resection (PPPD/Whipple) was performed in 15 cases (44.1%), total pancreatectomy in 14 cases (41.2%), and distal pancreatectomy in 5 cases (14.7%). Assumptions of vascular structure encasement (based on preoperative CT) revealed a different picture upon exploration. Although preoperative CT indicated SMA encasement in 36.7% (n=15), only 11.8% (n=4) of the nCTxR cohort exhibited SMA involvement requiring reconstruction. In contrast, preoperative CT demonstrated involvement of the coeliac trunk in 23.5% (n=8); ultimately, vascular replacement was necessary in 10 cases (29.4%) of the nCTxR cohort. (**Table 2**).

### Primary endpoints

#### Pre-chemotherapy CA19–9 level

Pre-CTx CA19–9 levels showed a noticeable difference between the two cohorts. The nCTxR cohort had a median level of 240 U/ml (IQR 38.8–1103 U/ml), compared to 635 U/ml (IQR 110–2092 U/ml) in the nCTxU cohort. However, this difference was slightly below the threshold of statistical significance (p = 0.15). (**Table 3**)

**Table 3:** CA 19–9 levels prior to naCTx and surgery, as well as calculated CA 19–9 ratios, were assessed.

	Overall	nCTxR	nCTxU	P
CA19–9 pre CTx [U/ml (IQR)]	475 (47 - 1359)	240 (38.8 - 1103)	635 (110 - 2092)	0.15
CA19–9 pre OP [U/ml (IQR)]	112 (23.6 – 401)	24 (4 – 126)	267 (42 – 1500)	**0.001**
CA19–9 Ratio [% (IQR)]	23.8 (10.5 -184)	11 (6 – 73)	85.4 (17.8 - 322)	0.07
CA19–9 level pre OP depending on chemotherapy protocol [U/ml (IQR)]				
Gemcitabine (+)	127 (8 – 555)	23.2 (5.6 – 105.8)	396 (79 – 1323)	0.07
FOLFOX/IRI	112 (38 – 395)	51.2 (7 – 103)	332 (110 – 1661)	0.25
CA19–9 ratio depending on chemotherapy protocol [% (IQR)]				
Gemcitabine (+)	25 (10 – 1885)	17 (6 – 76)	54 (14 – 671)	0.094
FOLFOX/IRI	20 (10 – 201)	11 (4 – 46)	123 (19 – 325)	0.3

Additionally, preoperative CA 19–9 levels and CA 19–9 ratios were stratified according to the different chemotherapy regimens. Bold values indicate statistical significance (p < 0.05).

#### Preoperative CA19–9 level

The preoperative CA19–9 levels differed significantly between the nCTxR cohort (24 U/ml (IQR 4–126 U/ml)) and the nCTxU (267 U/ml (IQR 42–1500 U/ml) (p = 0.001). Notably, these findings remain consistent across different chemotherapy protocols. Patients treated with Gemcitabine ± Nab-Paclitaxel/Cisplatin exhibited comparable trends, with a median preoperative CA19–9 level of 23.2 U/ml (IQR 5.6–105 U/ml) in the nCTxR cohort versus 396 U/ml (IQR 76–1323 U/ml) in the nCTxU cohort (p = 0.07).

Similarly, patients treated with FOLFOX/IRI had a median preoperative CA19–9 level of 51.2 U/ml (IQR 7–103 U/ml) in the nCTxR group, compared to 332 U/ml (IQR 110–1661 U/ml) in the nCTxU cohort (p = 0.25). (**Table 3**)

#### The pre - to post-chemotherapy CA19–9 ratio

The CA19–9 ratio between the pre-chemotherapy and pre-operation levels was calculated to evaluate the predictive value of the dynamic CA19–9 changes on the resectability status. Patients in the nCTxR group were found to retain a substantial lower CA 19–9 value with only 11% (IQR 6 – 73%) of their pre-chemotherapy levels compared to the nCTxU cohort with 85% (IQR 17.8 – 322%) (p = 0.07). Interestingly, patients undergoing neoadjuvant treatment with Gemcitabine ± Nab-Paclitaxel/Cisplatin, those classified as nCTxR achieved a median decrease in preoperative CA19–9 levels to 17% (IQR 6 – 76%), whereas nCTxU patients exhibited an increase by 54% (IQR 14 – 671%) of CA19–9 during chemotherapy (p = 0.09). Similar trends were observed in patients treated with FOLFOX/IRI. Here, nCTxR patients demonstrated a median reduction to 11% (IQR 4 – 46%) of baseline CA19–9 levels, while nCTxU patients showed a median reduction to only 23.5% (IQR 19 – 325%) (p = 0.3). (**Table 3**)

#### Predictive value of the pre-chemotherapy and pre-operative CA 19-9

To perform univariate and multivariate regression analyses, we defined cut-off values using receiver operating characteristic (ROC) curve analysis and corresponding AUC values (see **Supplementary Table 1**). The ROC-AUC Analysis revealed for the pre-chemotherapy CA 19–9 a cut-off value of 405 U/ml; (AUC 0.64), for the preoperative CA 19–9 a cut-off value of 105 U/ml; (AUC 0.85) and for the pre - to post-chemotherapy CA19–9 Ratio a cut of value of ≤ 15% (AUC 0.77).

Employing a univariate regression analysis, the pre-chemotherapy CA19–9 levels convincingly predicted the resection status. If the preCTx CA19–9 levels were below 450 U/ml, the OR for a resection following naCtx was 2.9 (95% CI: 1.04 – 8.52; p = 0.04). Likewise, preoperative CA19–9 levels below 105 U/ml increased the likelihood of curative intended resection with an OR of 13.9 (95% CI: 3.88 – 49.72; p = 0.001).

In the multivariate regression analysis, the preoperative CA19–9 level below the cut-off was identified as an independent predictive factor for resectability, with an OR of 13.8 (95% CI: 1.07 – 179; p = **0.04**). However, the pre-chemotherapy CA19–9 level below the cut-off lost its significance as a predictor for curative resection, with an OR of 1.7 (95% CI: 0.096 – 30.2; p = 0.71). The detailed results are presented in **Table 4**.

**Table 4:** Uni- and multivariate analysis for preCTx and preOP factors that may be associated with improved resectability.

	Univariate analysis			Multivariate analysis		
	Relative OR	95% CI	P	Relative OR	95% CI	P
CA19–9 pre CTx	2.9	1.04 – 8.53	**0.04**	1.7	0.096 – 30.26	0.718
CA19–9 pre OP	13.9	3.88 – 49.72	**0.001**	13.8	1.07 – 179.5	**0.044**
Ratio CA19-9	9	2.48 – 32.70	**0.001**	0.74	0.084 – 6.55	0.78
CEA pre CTx	1.5	0.49 – 4.55	0.474	**n.a**		
CEA pre OP	2.4	0.78 – 7.39	0.127	**n.a**		
Ratio CEA	2.3	0.62 – 8.82	0.2	**n.a**		
Plates pre CTx	2.6	0.88 – 7,78	0.083	**n.a**		
Plates pre OP	2.5	0.94 – 6.82	0.066	10.5	0.78 – 141.1	0.076
Ratio Plates	1.3	0.42 – 3.74	0.678	1	0.113 – 9.02	0.99
ALAT pre CTx	3.2	1.04 – 9.85	0.43	**n.a**		
ALAT pre OP	4	1.44 – 11.24	**0.008**	**n.a**		
Ratio ALAT	1.3	0.36 – 4.31	0.724	**n.a**		
ASAT pre CTx	5.5	1.61 – 18.26	**0.006**	**n.a**		
ASAT pre OP	4.1	1.46 – 11.57	**0.008**	14.8	0.95 – 232.3	0.054
Ratio ASAT	1.06	0.31 – 3.57	0.927	1.6	0.24 – 10.41	0.621
CA19-9>105U/ml; Ratio >15%	Reference			**n.a**		
CA19-9<105 U/ml; Ratio ≤15%	63	7.84 – 506.48	**0.001**	**n.a**		
CA19-9<105 U/ml; Ratio >15%	24	3.37 – 178	**0.002**	**n.a**		
CA19-9>105 U/ml; Ratio ≤15%	7	0.7 - 70	0.098	**n.a**		

Bold values indicate statistical significance (p < 0.05).

#### Predictive value of the pre - to post-chemotherapy CA 19–9 ratio

The positive CA19–9 pre - to post-chemotherapy ratio, where a reduction to ≤ 15% of the original CA19–9 value was seen after naCTx, increased the likelihood of resection in the univariate regression by an OR of 9 (95% CI: 2.4 – 32.7; p = **0.001**). Following the multivariate analysis, the CA19–9 ratio lost its significance as a predictive factor. (**Table 4**)

#### Resection rate after naCTx depends on CA19–9 dynamic

**Figure 2:**
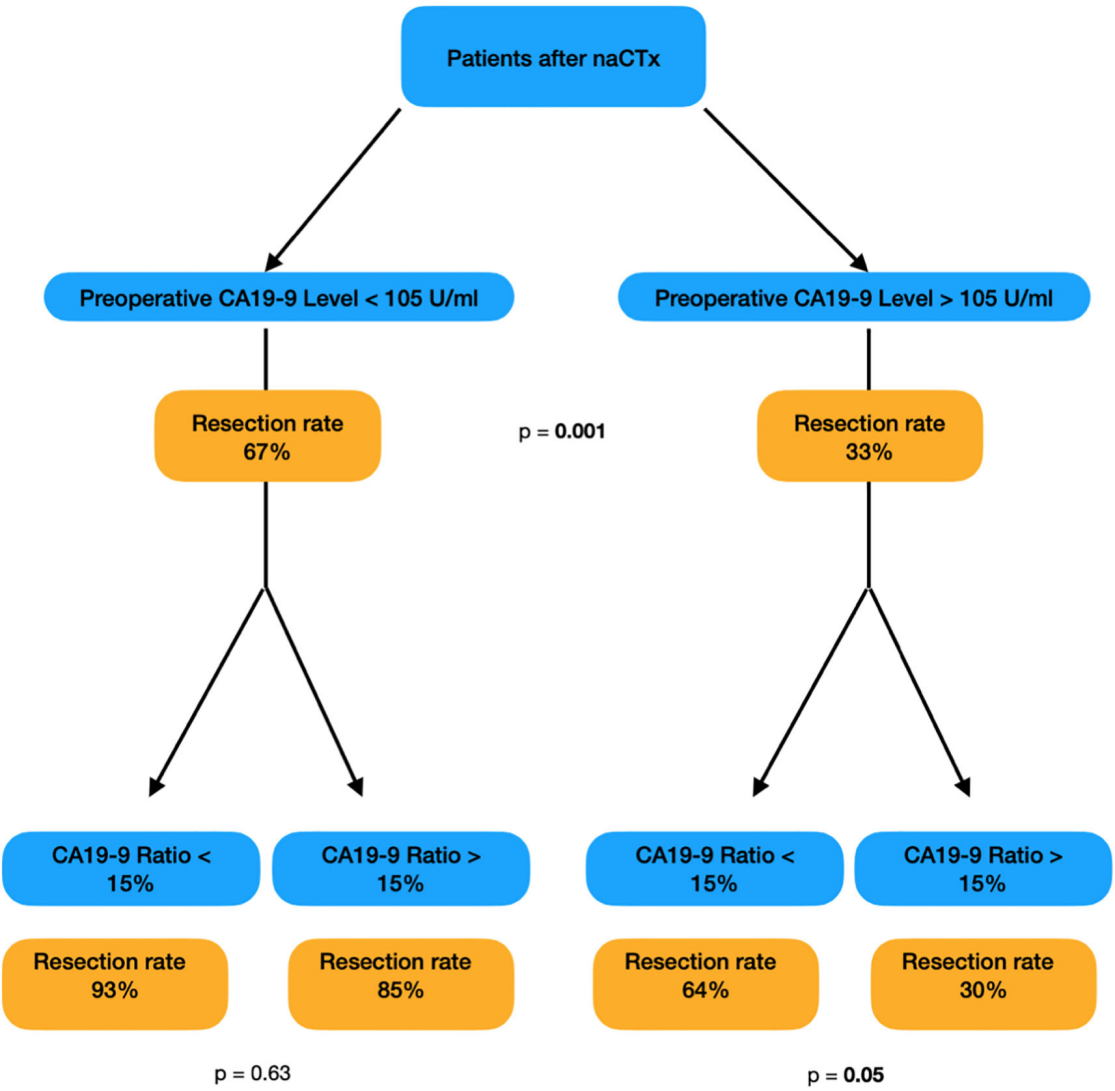
Resection rate calculated by the combination of preoperative CA19-9 value and the CA19-9 ratio.

As described above, both a preoperative CA19–9 level < 105 U/ml and a residual CA19–9 of ≤ 15% of the baseline are independent predictors of resection. If these two parameters are combined, the predictive accuracy increases dramatically. Patients with a preoperative CA19–9 Level below 105 U/ml were resected with a probability of 67%while Patients with *both*, CA19–9 levels below the cutoff of 105 U/ml and ≤ 15% from baseline value, had a resection rate of 93%. These results are presented in **Figure 2**.

#### Predictive accuracy is enhanced by combining preoperative CA19–9 levels with the CA19–9 ratio

For patients where the preoperative CA19–9 is < 105 U/ml and the residual CA19–9 is ≤ 15% the OR for curative resection is 63 (95% CI: 7.83 – 506.4; p = 0.001). Interestingly, if only the CA19–9 ratio is > 15%, the odds of being deemed resectable decrease to an OR of 24 (95% CI: 3.37 – 178; p = 0.002). (**Table 4**)

### Secondary endpoint

The median postoperative survival was 16.4 months (IQR 5.7 – 28.0 months) in the nCTxR group and 6 months (IQR 1.3 – 29.0 months) in the nCTxU group. This result approaches statistical significance (p = 0.06). The length of hospital stay was significantly shorter in the nCTxU group compared to the nCTxR group, with a median of 11 days (IQR 7 – 16.5 days) in the nCTxU group versus 17 days (IQR 14 – 25.5 days) in the nCTxR group (p = 0.02). A similar trend was observed in the occurrence of complications. CDC grade ≥ 3 complications occurred in 3 patients (8.8%) in the nCTxU group and in 9 patients (26.5%) in the nCTxR group (p = 0.092).

Overall, complications were evenly distributed between both groups (p = 0.385). However, postoperative bleeding occurred exclusively in the nCTxR group (n=4; 11.8%). In our entire cohort, 5 patients (7.4%) died during their hospital stay following surgery. See further information in **Table 5**.

**Table 5:** Secondary endpoints.

	Overall	nCTxR	nCTxU	P
Overall Survival ([months] (IQR))	20 (9.6 – 34.7)	22.8 (10.7 – 32.9)	11.8 (8.1 – 39)	0.16
Survival post OP ([months] (IQR))	9.67 (2.3 – 27.5)	16.4 (5.7 – 28)	6.06 (1.3 – 29)	0.057
CDC≥3 [n (%)]	12 (17.6)	9 (26.5)	3 (8.8)	0.092
Bleeding Event	4 (6.3)	4 (11.8)	0(0)	0.385
Anastomotic leak	4 (6.3)	2 (5.9)	2 (5.9)	
Others	4 (6.3)	3 (8.8)	1 (2.9)	
Time in Hospital ([days] (IQR))	14 (10 - 23)	17 (14 – 25.5)	11 (7 – 16.5)	**0.019**
In Hospital Mortality [n (%)]	5 (7.4)	3 (8.8)	2 (5.9)	0.667
Readmission to the OR [n(%)]	12 (18.8)	8 (23.5)	4 (11.8)	0.203

Overall survival shows almost significant differences. Postoperative complications and mortality did not differ between both groups. Bold values indicate statistical significance (p < 0.05).

## Discussion

The aim of this study was to evaluate paraclinical parameters that assist in predicting the resectability status of BR PDAC following naCTx. As surgical intervention remains the only curative option for this malignancy, and with its incidence expected to increase in the coming decades, optimizing preoperative assessment strategies is critically important.

One potential predictive biomarker is CA19-9. Some studies have reported an association between changes in CA19–9 levels during neoadjuvant therapy (CTx/RCTx), successful R0 resections and survival outcomes (25–28). For instance, Abbas et al. analyzed 250 patients with neoadjuvant-treated PDAC and demonstrated that a reduction of ≥85% in CA19–9 levels from baseline following neoadjuvant therapy significantly improved overall survival (23).

The unique aspect of our study is that it directly compares resected and unresected patients and focuses on the resection rate based on their pre-to-post chemotherapy course of the CA 19-9. Additionally, propensity score matching (PSM) between these groups further increases the robustness of our findings. Our study specifically focuses on patients with only neoadjuvant chemotherapy, whereas most existing literature combines radiotherapy (RTx) and chemotherapy (CTx).

We investigated CA19–9 levels prior to chemotherapy and prior to the operation and defined a preoperative CA19–9 cut-off value of < 105 U/ml and a dynamic reduction threshold of ≤ 15% from baseline CA19–9 levels as predictive for resectability. Notably, we combined these two parameters which showed a highly effective selection of patients who are resectable: We were able to show a resection rate of 93% for patients with both, a CA19–9 level below 105 U/ml and a reduction to ≤ 15% of the baseline level. Conversely, patients with both, a CA 19–9 level above the cutoff and a CA 19–9 reduction below the threshold resulted in a resection rate of only 30%.

As our data suggest, resectability is largely influenced by factors such as vascular infiltration, liver metastases, and peritoneal carcinomatosis. However, as previously noted, imaging modalities provide only a limited degree of accuracy in predicting tumor resectability (17, 29). The development and validation of robust biomarkers, along with clearly defined and clinically meaningful cut-off values, are essential to improve patient stratification and support personalized treatment planning.

In particular, such biomarker-based models may prove especially valuable in cases where radiologic findings are inconclusive. In light of these limitations, there is a growing emphasis on the integration of biomarkers into preoperative assessment. They could enable the identification of patients who might benefit from a diagnostic staging laparoscopy prior to definitive surgery. A study by Jambor et al. revealed that in 25% of patients with locally advanced pancreatic cancer, laparoscopy identified macroscopic or cytological metastases that were undetectable through imaging (18). This minimally invasive approach has proven particularly useful in accurately assessing the presence of liver metastases and peritoneal carcinomatosis, offering a more refined method for determining tumor resectability while reducing the associated procedural risks. As abdominal exploration in patients with PDAC carries a significant mortality risk of 16% exploratory laparoscopy emerges as a safer alternative, with a significant lower mortality (30).

Given the rate of major postoperative complications exceeding 14% in our cohort, exploratory laparotomy represents a non-negligible surgical intervention associated with considerable morbidity. In light of the urgent need to initiate palliative chemotherapy, the potential benefit of employing exploratory laparoscopy as a less invasive alternative in this clinical context warrants prospective investigation.

Our findings may contribute to enhancing the intraoperative decision-making. This is critical for making timely intraoperative decisions, as a prompt determination of unresectability reduces postoperative complication rates by minimizing surgical trauma and ensuring rapid recovery and early discharge (31). Particularly palliative patients will benefit from an early admission to palliative chemotherapy as survival significantly improves, when chemotherapy is admired early (32).

In addition, our data reflect the current standard of care in both Europe and Germany, where — based on the findings of the ESPAC5 trial — neoadjuvant treatment for BR PDAC is exclusively carried out using systemic chemotherapy, with radiotherapy playing no significant role in clinical practice (15, 22). The ESPAC5 trial found no significant superiority between these two chemotherapy regimens regarding resectability (15). Interestingly our analysis observed similar reductions of CA19–9 levels for FOLFOX/IRI and Gemcitabine ± Nab-Paclitaxel/Cisplatin, which will promote the finding of the ESPAC5 trail.

While our data raise the hypothesis that patients with an unfavorable CA19–9 dynamic might benefit from staging laparoscopy, this approach requires validation in prospective studies before being integrated into routine clinical decision-making.

Despite the retrospective nature of our study and the limited post-matching sample size (n = 34 per group), propensity score matching was applied to reduce confounding factors influencing CA19–9 levels, thereby enhancing the internal validity of our results. However, we acknowledge that the small cohort size may limit statistical precision, as reflected by wide confidence intervals. Additionally, with a broad range of indications, including patients witch venous and arterial resection we are well aware of the fact that this cohort and the conclusions drawed, might lack generalizability. Results of this analysis might be not easily applied in different centers.

Furthermore, CA19–9 non-producers were not excluded in this trial, which could have introduced additional variability. These limitations should be considered when interpreting our findings, and validation in larger prospective studies is warranted.

## Conclusion

Our study was able to identify two key criteria that may assist in the clinical routine to better distinguish non-resectable BR PDAC. Based on our findings in uni- and multivariate analysis, we can conclude that patients with a preoperative CA19–9 level of less than 105 U/ml, and a reduction of the baseline CA19–9 to ≤ 15% have the best chances for an R0 resection and an improved overall survival. When these criteria were met, we observed a resection rate of 93%, compared to only 30% when the criteria were not fulfilled.

CA19–9 monitoring during neoadjuvant chemotherapy may offers valuable insights into treatment efficacy and could serve as a complementary tool for staging evaluations during therapy. Further investigation into its integration with staging computed tomography is warranted to refine patient management strategies. However, further prospective studies with larger patient cohorts are needed to potentially develop an assessment tool to enhance the prediction of resectability in BR PDAC.

## Data Availability

The datasets generated and analyzed during the current study are not publicly available due to patient confidentiality and GDPR regulations but are available from the corresponding author on reasonable request.
