# Supplementary material for: Predicting resectability after neoadjuvant chemotherapy for patients with borderline resectable pancreatic cancer: a single center, retrospective trial
Source: Front Oncol. 2025 Sep 19;15:1602933. doi: 10.3389/fonc.2025.1602933 (PMC12490984; doi:10.3389/fonc.2025.1602933)
Supplement: Supplementary file 1 [file DataSheet1.pdf]

	Cut-off value	AUC	Sensitivity	1-Specificity
CA19-9 pre CTx	450 U/ml	0.64	0.69	0.29
**CA19-9 pre OP**	**105 U/ml**	**0.85**	**0.82**	**0.18**
**Pre-to-post chemotherapy CA19-9 ratio**	**15%**	**0.77**	**0.83**	**0.94**
CEA pre CTx	3 ng/ml	0.64	0.6	0.47
CEA pre OP	2.9 ng/ml	0.6	0.62	0.4
Ratio CEA	96%	0.57	0.61	0.47
Plates pre CTx	249 GPT/l	0.65	0.64	0.44
Plates pre OP	209 GPT/l	0.62	0.64	0.4
Ratio Plates	75%	0.46	0.6	0.65
ALAT pre CTx	0.55 µmol/l	0.66	0.67	0.35
ALAT pre OP	0.44 µmol/l	0.74	0.7	0.35
Ratio ALAT	27%	0.46	0.74	0.69
ASAT pre CTx	0.51 µmol/l	0.64	0.73	0.38
ASAT pre OP	0.5 µmol/l	0.7	0.65	0.19
Ratio ASAT	52%	0.4	0.65	0.6

Table 1, *Supplementary Data: Cut-off values and corresponding AUCs used for regression analyses are provided.*
